# Activation of a Cell Surface Signaling Pathway in *Pseudomonas aeruginosa* Requires ClpP Protease and New Sigma Factor Synthesis

**DOI:** 10.3389/fmicb.2017.02442

**Published:** 2017-12-12

**Authors:** Thomas F. Bishop, Lois W. Martin, Iain L. Lamont

**Affiliations:** Department of Biochemistry, University of Otago, Dunedin, New Zealand

**Keywords:** ECF sigma factor, antisigma, ClpP protease, pyoverdine, siderophore, regulated proteolysis, bacterial signal transduction, cell surface signaling

## Abstract

Extracytoplasmic function (ECF) sigma factors control expression of large numbers of genes in bacteria. Most ECF sigma factors are inhibited by antisigma proteins, with inhibition being relieved by environmental signals that lead to inactivation of the antisigma protein and consequent sigma factor activity. In cell surface signaling (CSS) systems in Gram negative bacteria antisigma activity is controlled by an outer membrane protein receptor and its ligand. In *Pseudomonas aeruginosa* one such system controls expression of genes for secretion and uptake of a siderophore, pyoverdine. In this system the activities of two sigma factors σ^FpvI^ and σ^PvdS^ are inhibited by antisigma protein FpvR_20_ that binds to the sigma factors, preventing their interaction with core RNA polymerase. Transport of ferripyoverdine by its outer membrane receptor FpvA causes proteolytic degradation of FpvR_20_, inducing expression of σ^FpvI^- and σ^PvdS^-dependent target genes. Here we show that degradation of FpvR_20_ and induction of target gene expression was initiated within 1 min of addition of pyoverdine. FpvR_20_ was only partially degraded in a mutant lacking the intracellular ClpP protease, resulting in an FpvR_20_ subfragment (FpvR_12_) that inhibited σ^FpvI^ and σ^PvdS^. The translation inhibitor chloramphenicol did not prevent induction of an σ^FpvI^-dependent gene, showing that degradation of FpvR_20_ released pre-existing σ^FpvI^ in an active form. However, chloramphenicol inhibited induction of σ^PvdS^-dependent genes showing that active σ^PvdS^ is not released when FpvR_20_ is degraded and instead, σ^PvdS^ must be synthesized in the absence of FpvR_20_ to be active. These findings show that sigma factor activation occurs rapidly following addition of the inducing signal in a CSS pathway and requires ClpP protease. Induction of gene expression that can arise from release of active sigma from an antisigma protein but can also require new sigma factor synthesis.

## Introduction

**FIGURE 1:**
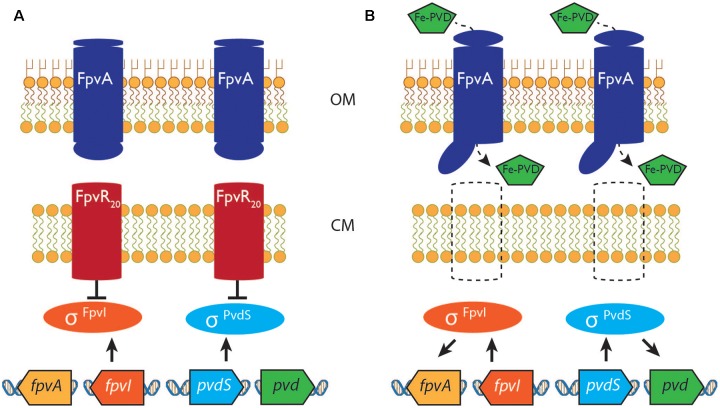
The pyoverdine signaling pathway. **(A)** In the absence of ferripyoverdine the activities of sigma factors σ^FpvI^ and σ^PvdS^ are inhibited by the antisigma protein FpvR_20_. **(B)** Import of ferripyoverdine (Fe-PVD) causes a molecular rearrangement of the FpvA receptor protein, triggering a proteolytic cascade that degrades FpvR_20_. σ^FpvI^ and σ^PvdS^ then become active, stimulating expression of the *fpvA* gene and of pyoverdine (pvd) synthesis genes, respectively. See text and (Llamas et al., 2014) for more detailed information. OM, outer membrane; CM, cytoplasmic membrane.

Extracytoplasmic function sigma factors are the largest and most diverse family of sigma factors in bacteria, directing expression of genes in response to a wide range of environmental stimuli (Staron et al., 2009; Ho and Ellermeier, 2012; Mascher, 2013). The activities of most ECF sigma factors are controlled by antisigma proteins that bind to and inhibit their cognate sigma factors (Campbell et al., 2007; Osterberg et al., 2011). CSS systems control the activities of a large proportion of the ECF sigma factors in Gram negative bacteria. In CSS systems antisigma protein activity (and hence that of the cognate ECF sigma factor) is controlled by an outer membrane protein receptor in response to an extracellular chemical signal, commonly a ferrisiderophore (Visca et al., 2002; Braun et al., 2006; Llamas et al., 2014). One of the best-characterized CSS systems controls expression of genes for synthesis of a siderophore pyoverdine and subsequent uptake of ferripyoverdine in the opportunistic pathogen *Pseudomonas aeruginosa* (**Figure 1**). In this system sigma factors σ^FpvI^ and σ^PvdS^ are inhibited by antisigma protein FpvR_20_ that is formed by cleavage of a 37 kDa precursor protein (Draper et al., 2011). FpvR_20_ extends from the periplasm through the cytoplasmic membrane into the cytoplasm and inhibition involves binding of the sigma factors by FpvR_20_, which also causes degradation of σ^PvdS^ although not σ^FpvI^ (Spencer et al., 2008; Edgar et al., 2014, 2017). Importation of ferripyoverdine results in molecular rearrangement of its receptor, FpvA (Schalk et al., 2009), initiating a proteolytic cascade that results in complete degradation of FpvR_20_. σ^FpvI^ and σ^PvdS^ then direct expression of genes for synthesis of FpvA and pyoverdine, respectively. σ^PvdS^ also directs expression of genes encoding a secreted exotoxin and a protease (Lamont et al., 2002). The rate of induction of target gene expression in response to the appropriate environmental signal has not been determined for this or any other CSS pathway.

The molecular mechanisms underlying signal transduction in CSS pathways are only partially understood. The proteolytic cascade that leads to degradation of FpvR_20_ includes the cytoplasmic membrane protease RseP but the other proteases involved have not yet been identified (Draper et al., 2011). RseP and its homologs are also required for cleavage of other antisigma proteins that inhibit ECF sigma factors (King-Lyons et al., 2007; Draper et al., 2011; Damron and Goldberg, 2012; Barchinger and Ades, 2013). The periplasmic protease Prc is part of the proteolytic cascade in other CSS systems (Bastiaansen et al., 2014) but is not required for signal transduction in the pyoverdine system (Draper et al., 2011). The protease(s) required for degradation of the cytoplasmic antisigma component and consequent sigma factor activity are not yet known in this or any other CSS pathway.

The simplest model for induction of gene expression in sigma-antisigma systems is that degradation of antisigma protein releases active sigma factor that can then interact with core RNA polymerase to initiate transcription from target promoters. To the best of our knowledge this model has not been tested experimentally. A possible alternative mechanism, suggested by proteolysis of σ^PvdS^ in the presence of FpvR_20_, is that sigma factors are inactivated following binding by antisigma proteins and are only active when synthesized in the absence of the cognate antisigma.

The aims of the work described here were to investigate the time-course of degradation of FpvR_20_ and consequent induction of target gene expression in response to the ferripyoverdine inducing signal; to identify the protease responsible for degrading the cytoplasmic portion of FpvR_20_; and to investigate whether active σ^PvdS^ and σ^FpvI^ sigma factors are released following proteolysis of FpvR_20_, or whether sigma factors must be synthesized in the absence of FpvR_20_ to be active.

## Materials and Methods

### Growth of Bacteria

Strains of *P. aeruginosa* used in this study are listed in **Table 1**. Bacteria were routinely grown in LB medium or on LB agar at 37°C. For Western blotting and reverse transcription quantitative PCR (RT-qPCR) *P. aeruginosa* was grown in King’s B medium (King et al., 1954). Antibiotics were added as required at the same concentrations as described previously (Mettrick and Lamont, 2009) with chloramphenicol being added to a final concentration of 150 μg/mL that completely prevents protein synthesis (Kay and Gronlund, 1969).

**Table 1:** Plasmids and strains of *Pseudomonas aeruginosa* used in this study.

Plasmids	Description	Reference
pEX18Gm	*oriT*^+^ *sacB*^+^ gene replacement vector; Gm^R^	Hoang et al., 1998
mini-CTX2	Tc^R^, integrating vector	Hoang et al., 2000
mini-CTX2*tigclpP*	Mini-CTX2 containing 2.3 kb fragment spanning *tig* and *clpP* genes (PAO1 genome region 1952446–1954761)	This study
pEX18Gm::Δ*clpP*	Allele replacement plasmid for the removal of PAO1 genome region 1954077–1954699, 0.9 and 0.8 kb fragments flanking *clpP* cloned into pEX18Gm as *Hin*dIII-*Bam*HI-*EcoR*I restriction fragments, Gm^R^	This study
pEX18Gm:: Δlon	Allele replacement plasmid for the removal of PAO1 genome region 1956251–1959420, 0.9 and 0.8 kb fragments flanking *lon* cloned into pEX18Gm as *Hin*dIII-*Bam*HI-*EcoR*I restriction fragments, Gm^R^	This study
Strains of *P. aeruginosa*		
PAO1	Wild-type; Pvd^+^	Stover et al., 2000
PAO1 *pvdF*	PAO1 *pvdF*::Km^R^; Pvd^-^	McMorran et al., 2001
PAO1 *pvdF clpP*	PAO1 *pvdF* with an unmarked *clpP* deletion	This study
PAO1 *pvdF clpP* (ctx::tig*clpP*)	PAO1 *pvdF clpP* containing mini-CTX2*tigclpP*	This study
PAO1 *pvdF lon*	PAO1 *pvdF* with an unmarked lon deletion	This study

### Genetic Manipulations

Plasmids used in this study are listed in **Table 1**. Restriction of DNA molecules and DNA cloning were carried out using standard methods (Sambrook et al., 2000) with enzymes purchased from Roche Molecular Biologicals. DNA fragments required for strain construction were amplified from genomic DNA of *P. aeruginosa* PAO1 by PCR with FirePol DNA Polymerase (Solis Biodyne) or Taq DNA Polymerase Reddymix (ThermoPrime) using appropriate primers (Supplementary Table S1) that were designed on the basis of the *P. aeruginosa* PAO1 genome sequence^1^ (Winsor et al., 2011). DNA fragments were cloned into the required vectors and all plasmid constructs were verified by DNA sequencing.

Construction of an unmarked deletion in the *P. aeruginosa clpP* gene was carried out as described previously (Hoang et al., 1998; Mettrick and Lamont, 2009; Draper et al., 2011). Briefly, fragments of DNA flanking the deletion site in *clpP* were amplified by PCR using primer pairs listed in Supplementary Table S1, ligated together and cloned into the allele replacement vector pEX18Gm to give plasmid pEX18Gm::Δ*clpP*. Chromosomal allele replacement was then carried out (Hoang et al., 1998). Over 90% of *clpP* was deleted and the deletion was in-frame with downstream genes. Deletions and allele replacements were confirmed by PCR. An analogous method was used to create a deletion in the *lon* gene. For complementation of the *clpP* mutation, a 2.3 kb PCR product spanning the *clpP* gene, the upstream *tig* gene and the predicted promoter was cloned into the integrating plasmid miniCTX2 (Hoang et al., 2000) that was then transferred into *P. aeruginosa pvdF clpP* by conjugation from *Escherichia coli* S17-1 as described previously (Shirley and Lamont, 2009).

### Western Blotting

Bacteria were grown in King’s B medium (King et al., 1954) (20 mL) to late exponential phase (OD_600_ between 1.8 and 2.2 [0.6 and 0.8 for PAO1 *pvdF clpP*]). A sample (400 μL [1000 μL for PAO1 *pvdF clpP*]) was centrifuged in a bench-top microcentrifuge (13,000 rpm, 20 s) and the pellet resuspended in SDS–PAGE loading buffer [2% (w/v) SDS, 5% (w/v) β-mercaptoethanol, 10% (v/v) glycerol, 0.002% bromophenol blue, 62.5 mM Tris–HCl; pH 6.8] (15 μL [8 μL for PAO1 *pvdF clpP*]) and PBS (85 μL [42 μL for PAO1 *pvdF clpP*]) at 99°C. Pyoverdine (150 μM), purified from *P. aeruginosa* PAO1 as described previously (Mettrick and Lamont, 2009), was added to each culture and samples were taken 1, 3, 5, 10, 20, 30, 60, 90, and 120 min after the addition of pyoverdine. The samples were centrifuged and the pellets resuspended as described above. The tubes were heated at 99°C for 20 min, centrifuged briefly and vortexed. Proteins were separated by electrophoresis on 12.5% SDS–PAGE gels and transferred to nitrocellulose membranes using standard methods (Harlow and Lane, 1999). Membranes were blocked in 15% (v/v) SeaBlock (Pierce) in TBS buffer (0.9% NaCl, 100 mM Tris–HCl; pH 7.5) containing 0.1% (v/v) Tween. Blots were probed with monoclonal antibody anti-σ^PvdS^ (Xiong et al., 2000) or anti-FpvRN (Draper et al., 2011) that was raised against a peptide corresponding to residues 62–75 within the N-terminal (cytoplasmic) region of FpvR_20_. Equal protein loadings were confirmed by probing the membranes with monoclonal antibody anti-RpoD (Santa Cruz Biotechnology). Detection was carried out using anti-Mouse HRP conjugates (Sigma), Super Signal ECL (Pierce), and a Fuji LAS-1000 Imager. Western blotting was carried out with bacteria from at least two independent cultures for each strain and growth condition and representative data are shown.

### RT-qPCR

Bacteria were grown at 37°C in King’s B medium (King et al., 1954) (20 mL) to late exponential phase. A sample (500 μL) was taken from each culture (0 min) and transferred to a tube containing 1 mL of RNAprotect Bacteria Reagent (Qiagen). Pyoverdine (150 μM) was added to each culture. Samples (500 μL) were taken 5, 10, 30, and 60 min after the addition of pyoverdine. The samples were transferred to tubes containing 1 mL of RNAprotect Bacteria Reagent (Qiagen) and vortexed vigorously. RNA was extracted and RT-qPCR, including control reactions without template or without reverse transcriptase, was carried out as described previously (Draper et al., 2011; Konings et al., 2013). Relative quantification was performed using the second derivative maximum method corrected for primer efficiencies, with *clpX* and *oprL* as the combined reference genes, as described previously (Draper et al., 2011; Konings et al., 2013); the reference genes gave similar outcomes when used individually. qPCR was carried out twice for each cDNA sample, with three replicates each time. cDNA was prepared from at least two independent cultures for each strain and growth condition and representative data are shown.

## Results

### Time Course for Induction of Gene Expression

**FIGURE 2:**
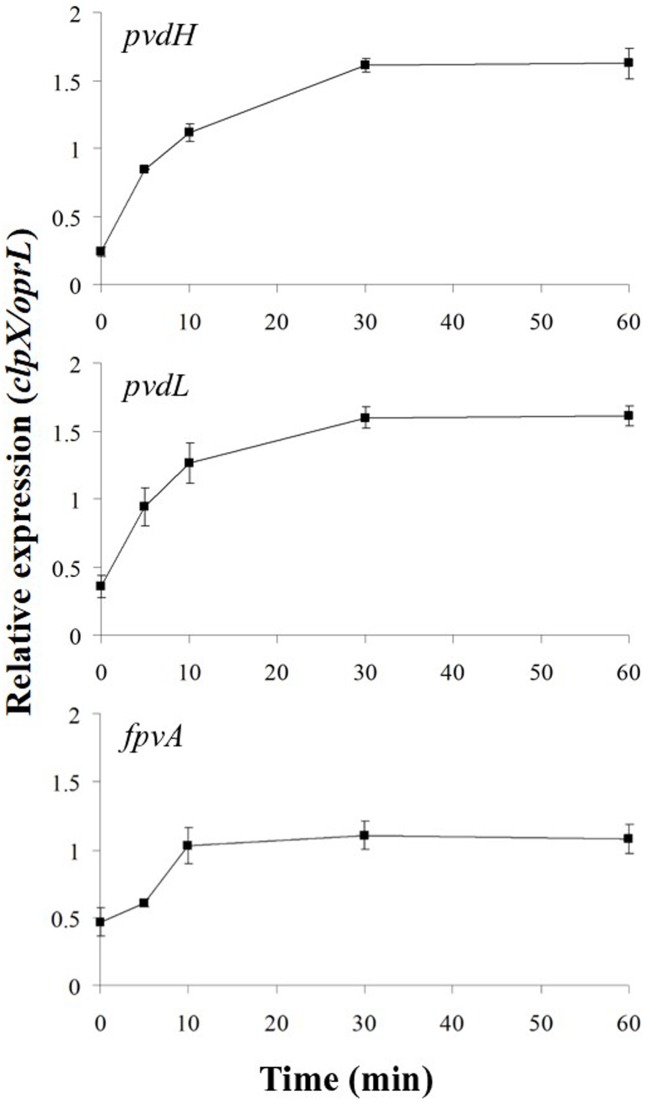
Activation of gene expression following addition of pyoverdine. Pyoverdine was added to *Pseudomonas aeruginosa* PAO1 *pvdF* bacteria (0 min) and samples were collected at intervals and analyzed by reverse transcription quantitative PCR (RT-qPCR). The amounts of *pvdH*, *pvdL*, and *fpvA* transcripts are shown relative to the reference genes *clpX* and *oprL*. Data are means of six technical replicates with standard deviation shown. Similar results were obtained when the experiment was repeated (Supplementary Figure S1).

In the absence of ferripyoverdine, FpvR_20_ inhibits σ^PvdS^ and σ^FpvI^. Addition of pyoverdine, which chelates iron to become ferripyoverdine, induces σ^PvdS^-dependent expression of pyoverdine synthesis genes and the σ^FpvI^-dependent gene *fpvA* (Lamont et al., 2002; Beare et al., 2003; Tiburzi et al., 2008; Draper et al., 2011). However, the rate of increase in gene expression following addition of the inducing signal has not been determined for this or any other CSS system. We therefore determined the time-course of activation of transcription for two σ^PvdS^-dependent genes *pvdH* and *pvdL*, and the sole known σ^FpvI^-dependent gene *fpvA* (**Figure 2** and Supplementary Figure S1). *pvdH* and *pvdL* had very similar rates of induction, with maximal transcription 30 min after addition of pyoverdine. Expression of the *fpvA* gene was also maximal after 30 min although transcription of this gene was less strongly induced than that of *pvdH* and *pvdL*.

### Degradation of FpvR_20_ and σ^PvdS^

**FIGURE 3:**
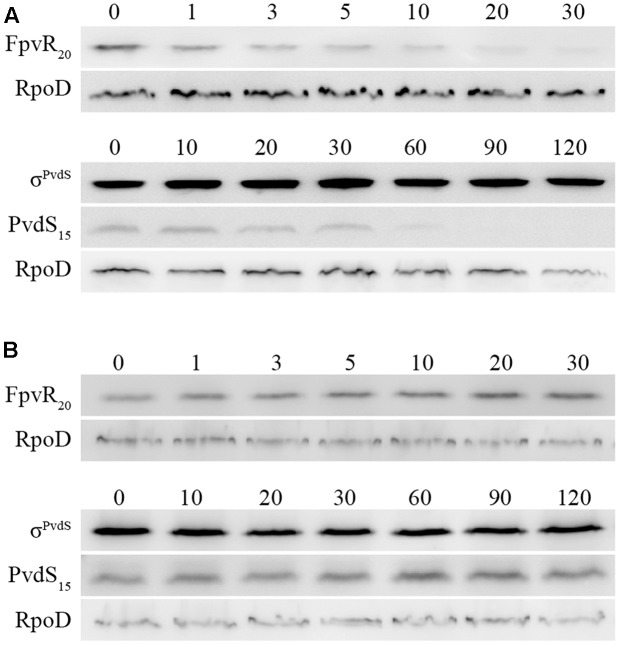
Time-course of degradation of FpvR_20_ and σ^PvdS^ following addition of pyoverdine. Pyoverdine was added to *P. aeruginosa* bacteria (0 min). Samples were collected at intervals and analyzed by Western blotting with antibodies against FpvR_20_, σ^PvdS^ or RpoD (loading control). FpvR_20_, σ^PvdS^ (full-size), PvdS_15_ and RpoD are indicated. Times are shown in minutes. **(A)** PAO1 *pvdF* **(B)** PAO1 *pvdF fpvA*. Similar results were obtained when the experiment was repeated (Supplementary Figure S2).

σ^PvdS^ and σ^FpvI^ are inhibited by the FpvR_20_ protein and induction of gene expression following addition of pyoverdine requires degradation of FpvR_20_ (Draper et al., 2011). To determine the time-course of degradation of FpvR_20_, Western blotting was carried out using an antibody specific to the cytoplasmic (sigma factor binding) domain of the protein (**Figure 3A** and Supplementary Figure S2). There was significantly less FpvR_20_ per cell within 1 min of addition of pyoverdine and FpvR_20_ was almost undetectable by 30 min. This timeframe correlates well with the induction of gene expression (**Figure 2**). Pyoverdine-mediated induction of gene expression is dependent on the FpvA ferripyoverdine receptor protein (Shen et al., 2002; Beare et al., 2003; James et al., 2005; Draper et al., 2011). Addition of pyoverdine to an *fpvA* mutant did not result in degradation of FpvR_20_ (**Figure 3B** and Supplementary Figure S2) confirming the requirement for FpvA as well as pyoverdine for degradation of FpvR_20_.

The presence of FpvR_20_ results in proteolysis of σ^PvdS^ to generate a subfragment (PvdS_15_) that is likely to be an intermediate in the proteolytic degradation of σ^PvdS^, lowering the amount of σ^PvdS^ per cell (Spencer et al., 2008). The amount of PvdS_15_ decreased following the addition of pyoverdine (**Figure 3A**). In the absence of FpvA (and consequent presence of FpvR_20_) there was no change in the amount of PvdS_15_ following addition of pyoverdine (**Figure 3B**). These findings are consistent with the requirement of FpvR_20_ for degradation of σ^PvdS^ (Spencer et al., 2008). The amount of σ^FpvI^ per cell is not altered by the presence of FpvR_20_ (Edgar et al., 2017).

### ClpP Protease Is Part of the Degradation Pathway

**FIGURE 4:**
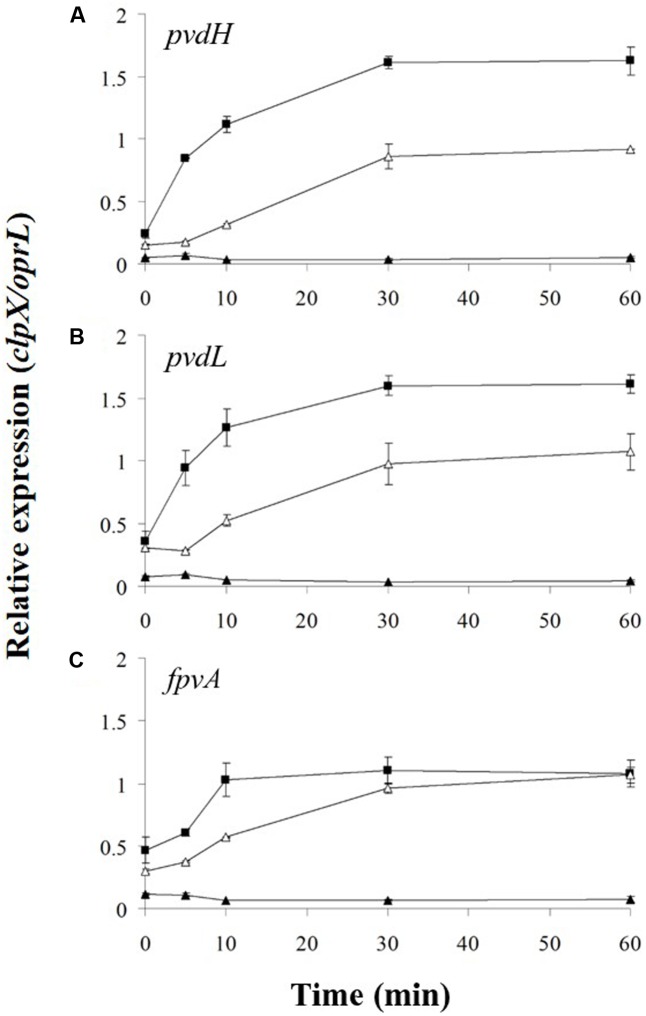
Effect of *clpP* mutation on induction of gene expression. Pyoverdine was added (0 min) to *P. aeruginosa* PAO1 *pvdF clpP* (black triangles) and *P. aeruginosa* PAO1 *pvdF clpP* (minictx::*tig-clpP*) (open triangles). Samples were collected at intervals and analyzed by RT-qPCR. Data are means of six technical replicates with standard deviation shown. Equivalent data from strain PAO1 *pvdF* bacteria (**Figure 2**) (black squares) are included for comparison. **(A)** *pvdH*. **(B)** *pvdL*. **(C)** *fpvA*.

Degradation of FpvR_20_ requires one or more cytoplasmic proteases (Draper et al., 2011). The cytoplasmic protease ClpP contributes to the degradation of the antisigma factors RseA in *E. coli* (Flynn et al., 2004) and RsiW in *Bacillus subtilis* (Zellmeier et al., 2006). We therefore tested the hypothesis that ClpP contributes to degradation of FpvR_20_ and consequent activity of σ^PvdS^ and σ^FpvI^. An in-frame deletion of the *clpP* (PA1801) gene was engineered in *P. aeruginosa* PAO1 *pvdF*. The effect of the *clpP* mutation on gene expression was then determined (**Figure 4** and Supplementary Figure S3). The *clpP* mutation completely prevented induction of expression of *pvdH*, *pvdL*, and *fpvA*. Indeed, gene expression in the *clpP* mutant was even lower than in Clp+ bacteria in the absence of pyoverdine. Complementation of the mutant with wild-type *clpP* restored gene expression. These data show that ClpP protease is essential for induction of gene expression in the pyoverdine signaling pathway.

**FIGURE 5:**
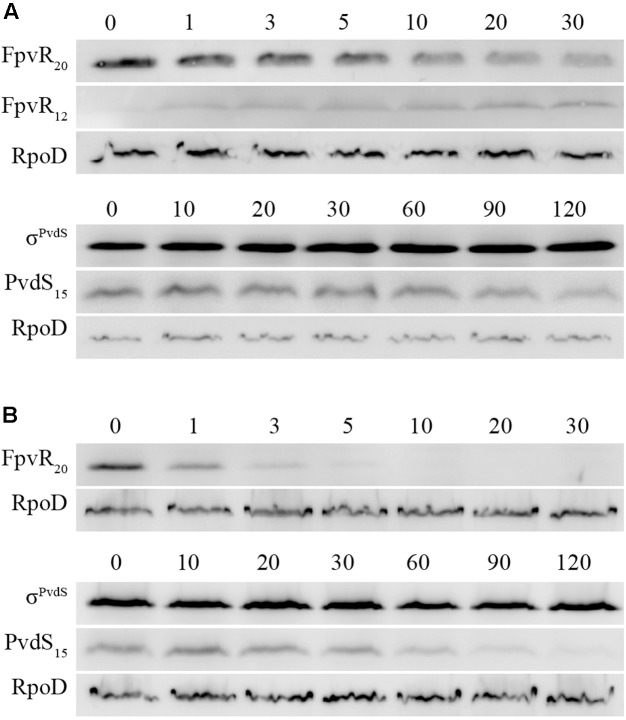
Effects of *clpP* mutation on degradation of FpvR_20_ and σ^PvdS^. Pyoverdine was added (0 min) to *P. aeruginosa* PAO1 *pvdF* bacteria containing a mutation in the *clpP* gene. Samples were collected at intervals and analyzed by Western blotting. Times are shown in minutes. **(A)** *P. aeruginosa* PAO1 *pvdF clpP*. **(B)** *P. aeruginosa* PAO1 *pvdF clpP* (minictx::*tig-clpP*). FpvR_20_, FpvR_12_, σ^PvdS^ (full-size), PvdS_15_ and RpoD are indicated. FpvR_12_ was not detected in *P. aeruginosa* PAO1 *pvdF clpP* (minictx::*tig-clpP*). Similar results were obtained when the experiment was repeated (Supplementary Figure S4).

We therefore tested the hypothesis that ClpP is required for degradation of FpvR_20_. Following addition of pyoverdine to *clpP* mutant bacteria, FpvR_20_ was degraded and a sub-fragment of approx. 12 kDa (FpvR_12_) was present that was not detected in Clp^+^ strains (**Figure 5A** and Supplementary Figure S4). The increasing amount of this fragment following addition of pyoverdine was inversely proportional to the decreasing amount of FpvR_20_ indicating that FpvR_12_ is formed as a result of proteolysis of FpvR_20_. The FpvR_12_ fragment retains the cytoplasmic sigma binding domain of FpvR_20_ (Edgar et al., 2014) explaining the inhibition of sigma factor activity that occurs in the *clpP* mutant even when pyoverdine is present (**Figure 4**). Complementation with wild-type *clpP* restored wild-type phenotype of an absence of FpvR_12_ (**Figure 5B** and Supplementary Figure S4) and largely restored expression of the *pvdH*, *pvdL*, and *fpvA* genes (**Figure 4**); incomplete restoration of gene expression was most likely a consequence of the different chromosomal context of the introduced *clpP* gene. In contrast to ClpP^+^ bacteria, reduction in the amount of PvdS_15_ following addition of pyoverdine did not occur in the *clpP* mutant (**Figure 5A** and Supplementary Figure S4). Collectively these data show that ClpP protease is required for, and most likely catalyzes, degradation of the cytoplasmic portion of FpvR_20_.

### Gene Expression Requires Newly Synthesized σ^PvdS^ But Not σ^FpvI^

The proteolytic degradation of σ^PvdS^ that occurs in the presence of FpvR_20_ raised the question, is active sigma factor released when FpvR_20_ is degraded or must the sigma factors be synthesized in the absence of FpvR_20_ to be active? To address this question, the effects of the translation inhibitor chloramphenicol on transcription of target genes were measured. If newly synthesized sigma factor is required for transcription of target genes, prevention of sigma factor synthesis would prevent increased target gene expression; however, if degradation of FpvR_20_ releases active sigma factor, chloramphenicol would not prevent induction of target gene expression.

**FIGURE 6:**
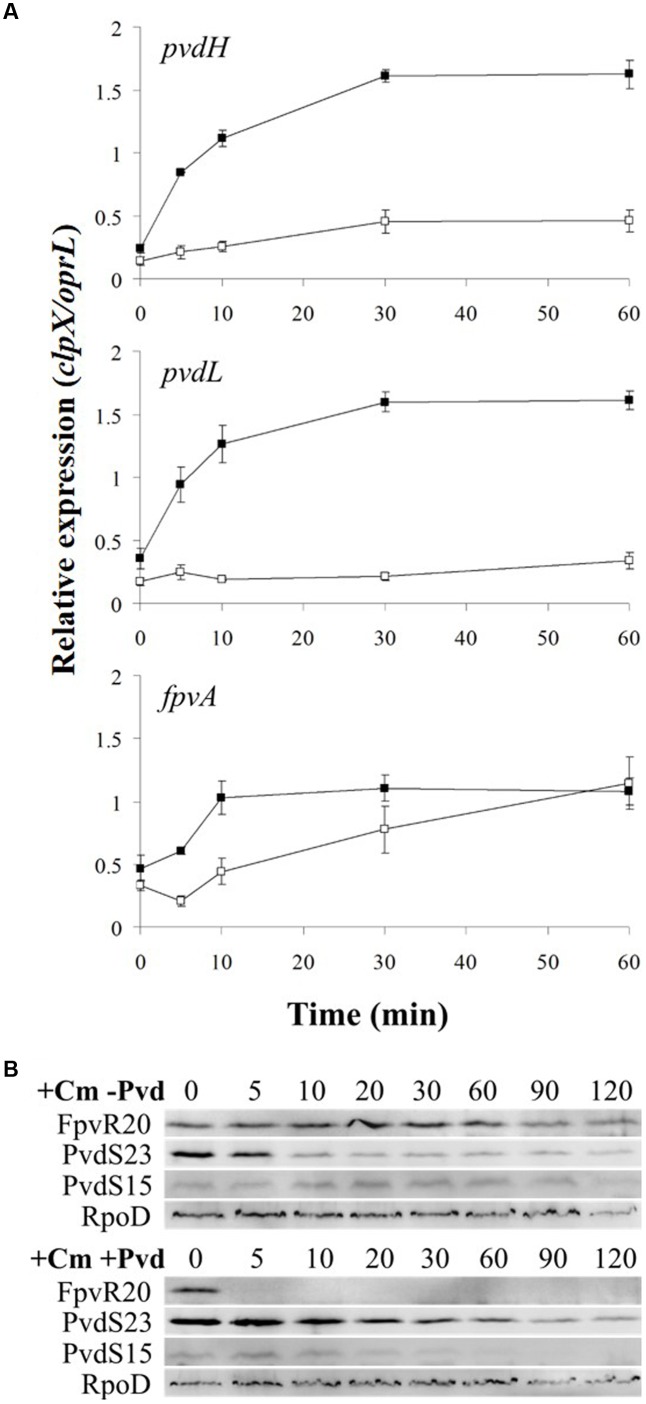
Effect of chloramphenicol on induction of gene expression. Chloramphenicol (Cm) and then pyoverdine were added to *P. aeruginosa* PAO1 *pvdF* bacteria (0 min) and samples were collected at intervals. **(A)** Samples were analyzed by RT-qPCR for *pvdH*, *pvdL*, and *fpvA*. Open squares, Cm present; Black squares, Cm absent. Data are means of six technical replicates with standard deviation shown. **(B)** Samples were analyzed by Western blotting for σ^PvdS^ or FpvR_20_ in the presence or absence of pyoverdine, as shown. FpvR_20_, σ^PvdS^ (full-size), PvdS_15_ and RpoD are indicated. Times are shown in minutes. Similar results were obtained when the experiment was repeated (Supplementary Figure S5).

The results are shown in **Figure 6A** and Supplementary Figure S5A. Addition of chloramphenicol prevented pyoverdine-mediated induction of expression of *pvdH* and *pvdL*, indicating that σ^PvdS^ must be synthesized in the absence of FpvR_20_ for *pvd* gene expression to occur. Induction of *fpvA* gene expression was not prevented by the presence of chloramphenicol, indicating that degradation of FpvR_20_ released active σ^FpvI^ that could direct transcription of *fpvA*. The slight delay in induction of expression of *fpvA* in the presence of chloramphenicol may indicate that σ^FpvI^ released from degraded FpvR_20_ would normally be supplemented by newly synthesized σ^FpvI^ during pyoverdine-mediated induction of gene expression.

The effect of chloramphenicol on protein amounts was also investigated (**Figure 6B**). In the presence of pyoverdine FpvR_20_ was rapidly degraded, as expected and as also occurred in the absence of chloramphenicol (**Figure 2**). In the absence of pyoverdine the amount of FpvR_20_ per cell did not greatly decrease for at least 60 min following addition of chloramphenicol. The continuing presence of FpvR_20_ in the absence of protein synthesis indicates that this protein is relatively stable under these conditions.

In the absence of pyoverdine and consequent presence of FpvR_20_, the amount of σ^PvdS^ decreased after the addition of chloramphenicol (**Figure 6B** and Supplementary Figure S5B). The amount of PvdS_15_ formed from σ^PvdS^ did not vary during the course of the experiment suggesting that the rates of formation and degradation of PvdS_15_ are similar. The decrease in the amount of σ^PvdS^ was slower following the addition of pyoverdine and consequent absence of FpvR_20_. These results indicate that FpvR_20_ accelerates, but is not essential for, degradation of σ^PvdS^.

## Discussion

In this research we show that degradation of the FpvR_20_ antisigma protein in the pyoverdine CSS pathway occurs within 1 min of addition of pyoverdine with expression of the *pvd* and *fpvA* target genes being maximal by 30 min. Target gene expression is dependent on ClpP protease for removal of FpvR_20_ antisigma activity and requires *de novo* synthesis of σ^PvdS^, but not σ^FpvI^.

The time-course of antisigma degradation has previously been examined for the *E. coli* stress response ECF sigma-antisigma system, in which antisigma RseA inhibits the activity of sigma factor σ^E^. RseA has a half-life of about 8 min in uninduced bacteria and 1–2 min following heat-induced envelope stress (Ades et al., 2003; Chaba et al., 2007). In the absence of the pyoverdine inducing signal there was no detectable reduction in the amount of FpvR_20_ over at least 60 min when chloramphenicol was present to prevent new protein synthesis (**Figure 6B**), indicating that FpvR_20_ has a long half-life under non-inducing conditions. In contrast, FpvR_20_ was almost undetectable 5 min after addition of pyoverdine in the presence of chloramphenicol (**Figure 6B**) indicating a much shorter half-life. The rates of degradation of the FpvR_20_ and RseA antisigma proteins in induced and uninduced cells are therefore comparable, in each case allowing a rapid response to the relevant environmental signal. So far as we are aware the time-course of induction of target gene expression has not previously been determined for any CSS system or indeed, any ECF sigma-antisigma system.

The proteolytic cascade that leads to degradation of FpvR_20_ and induction of gene expression requires ClpP protease (**Figures 4**, **5**). A CSS system is therefore part of the expanding repertoire of regulatory pathways in which Clp proteases are required for degradation of an antisigma protein and consequent sigma factor activity (Flynn et al., 2004; Zellmeier et al., 2006). Unfoldase chaperones – either ClpA or ClpX – are required to render substrate proteins susceptible to proteolysis by ClpP (Liu et al., 2014; Olivares et al., 2016) and it will be of interest to determine which unfoldase is required for complete degradation of FpvR_20_. In the *clpP* mutant a subfragment of FpvR_20_, FpvR_12_, is present that is presumably formed by proteolysis of FpvR_20_ and is further degraded by a ClpP-containing protease in ClpP^+^ bacteria. The size of FpvR_12_ indicates that it contains the complete cytoplasmic antisigma domain of FpvR_20_ and so inhibits σ^PvdS^ and σ^FpvI^ (Edgar et al., 2014, 2017). This is consistent with the finding that expression of *pvdH*, *pvdL*, and *fpvA* was significantly lower in the *clpP* mutant than in Clp^+^ bacteria in the absence of pyoverdine (**Figure 6**). Although FpvR_20_ has a long half-life in the absence of pyoverdine there was a gradual reduction in the amount of FpvR_20_ in the absence of new protein synthesis (**Figure 4**). These findings are consistent with proteolytic degradation of FpvR_20_ in wild-type bacteria even in the absence of pyoverdine. Auto-inducing systems such as the pyoverdine CSS system require a basal level of gene expression in order to produce and detect the inducing signal. In the pyoverdine system, some degradation of FpvR_20_ in the absence of pyoverdine is evidently necessary for low-level sigma factor activity to provide basal expression of *fpvA* and *pvd* genes that is needed for up-regulation of gene expression when pyoverdine is present.

The simplest model for sigma factor activation in sigma-antisigma systems is that degradation of the antisigma protein results in release of active sigma factor and consequent gene expression (Brooks and Buchanan, 2008; Ho and Ellermeier, 2012). So far as we are aware, this model has not been directly tested for any sigma/antisigma pair. Our data indicate that σ^PvdS^ must be synthesized in the absence of the FpvR_20_ antisigma in order for expression of *pvd* target genes to occur following addition of pyoverdine (**Figure 6**). This demonstrates an alternative model, in which a sigma factor must be synthesized in the absence of its cognate antisigma in order to be active. The presence of FpvR_20_ is associated with proteolysis of σ^PvdS^ that generates a subfragment (PvdS_15_), a likely intermediate in the proteolytic degradation of σ^PvdS^, resulting in a lower amount of σ^PvdS^ per cell when FpvR_20_ is present (**Figure 3**) (Spencer et al., 2008). Proteolysis of σ^PvdS^ in the presence of FpvR_20_ may partially explain the requirement for sigma factor synthesis in the absence of FpvR_20_ in order for the σ^PvdS^ to be active and for target gene expression to occur following addition of pyoverdine. Conversely induction of the σ^FpvI^-dependent *fpvA* gene did not require new protein synthesis (**Figure 4A**) showing that in this case, degradation of FpvR_20_ did result in release of active sigma factor. This is consistent with the cellular level of σ^FpvI^ being unaffected by the presence of FpvR_20_ (Edgar et al., 2017) indicating that FpvR_20_ does not trigger degradation of σ^FpvI^. It will be of interest to determine whether other ECF sigma factors must, like σ^PvdS^, be synthesized in the absence of the cognate anti-sigma in order to be active. A mutation in *clpP* or in the gene encoding Lon protease that degrades a number of regulatory proteins (Van Melderen and Aertsen, 2009) did not prevent formation of PvdS_15_ (**Figure 5** and Supplementary Figure S6). The protease that catalyzes the formation of PvdS_15_ from σ^PvdS^ therefore remains to be identified.

## Conclusion

Our results demonstrate the speed with which a CSS system can respond to the presence of the inducing signal and the importance of ClpP protease in this process. They also show that induction of gene expression in an ECF sigma-antisigma system can require synthesis of sigma factor in the absence of antisigma, rather than release of active sigma factor following degradation of the antisigma. The molecular mechanisms that initiate degradation of FpvR_20_ and presumably involve its interaction with FpvA remain to be elucidated.

## Author Contributions

IL conceived the study and the project outline. TB, LM, and IL designed methods and experiments, analyzed the data and interpreted results. TB and LM carried out experiments and collected data. IL, TB, and LM wrote the manuscript. IL, TB, and LM contributed to revision of, and approved, the final manuscript.

## Conflict of Interest Statement

The authors declare that the research was conducted in the absence of any commercial or financial relationships that could be construed as a potential conflict of interest.

## Footnotes

1 http://beta.pseudomonas.com

## Abbreviations

CSS: cell surface signaling
ECF: extracytoplasmic function
