# Supplementary material for: Activation of a Cell Surface Signaling Pathway in Pseudomonas aeruginosa Requires ClpP Protease and New Sigma Factor Synthesis
Source: Front Microbiol. 2017 Dec 12;8:2442. doi: 10.3389/fmicb.2017.02442 (PMC5733041; doi:10.3389/fmicb.2017.02442)
Supplement: Supplementary file 1 [file Table_1.PDF]

Supplementary Table

Activation of a Cell Surface Signaling Pathway in *Pseudomonas aeruginosa* requires ClpP Protease and New Sigma Factor Synthesis

Thomas F. Bishop, Lois W. Martin and Iain L. Lamont*

*** Correspondence:** Corresponding Author: iain.lamont@otago.ac.nz

Supplementary Table 1. Oligonucleotide primers used in this study

Primer	Primer Sequence (5' to 3')	Use and reference*
RTpvdLf	ACCCTGCGTGCTGATGTC	qPCR of *pvdL* (Konings et al., 2013)
RTpvdLr	TCGGCTCGGAACCGGAGAA	qPCR of *pvdL* (Konings et al., 2013)
RTpvdHf	CAGCACCATCCTGTCGTTCCA	qPCR of *pvdH* (Konings et al., 2013)
RTpvdHr	GCAGGTTCGCCTTGACCC	qPCR of *pvdH* (Konings et al., 2013)
RTfpvAf	CCAGCACCACACGGTCTCAG	qPCR of *fpvA* (Draper et al., 2011)
RTfpvAr	GCTACGCTTGTTCCGGACCTC	qPCR of *fpvA* (Draper et al., 2011)
RTclpXf	GTGGGCGAGGATGTCGAGAAC	qPCR of *clpX* (Draper et al., 2011; Konings et al., 2013)
RTclpXr	CGGTACCCTCGATGAGCTTCAG	qPCR of *clpX* (Draper et al., 2011; Konings et al., 2013)
RToprLf	CCAACAGCGGTGCCGTTGA	qPCR of *oprL* (Draper et al., 2011; Konings et al., 2013)
RToprLr	GCCATATTGTACTCGCGGGT	qPCR of *oprL* (Draper et al., 2011; Konings et al., 2013)
tigclpPfor	GGAAGCTTCATCGACTTCGTCGGCAAGATC	Deletion of *clpP* gene (this study)

tigclpPrev	GGGGATCCGCGAGACATGTCTTGCGATCAC	Deletion of *clpP* gene (this study)
clpPclpXrev	GGGAATTCCTGCGGATGCTTGCGAC	Deletion of *clpP* gene (this study)
clpPclpXfor	GGGGATCCCTGGCCGTCTAAGGCTCC	Deletion of *clpP* gene (this study)
tigclpPup	GGGACTAGTTCGGAGCGGAATCGGCTG	Complementation of *clpP* mutation (this study)
tigclpPdown	GGGTACCACGAGGCGGCCGAATGTC	Complementation of *clpP* mutation (this study)
lonupfor	GGGAAGCTTCGATGTGGAGAAGGCCCAGATG	Deletion of *lon* gene (this study)
lonuprev	GGGGGATCCGTCACGTAGCGGCAGCAAG	Deletion of *lon* gene (this study)
lonhupfor	GGGGATCCAGCACGCATTAGTCGGTAGG	Deletion of *lon* gene (this study)
lonhuprev	GGGAATTCCGGCCTGTTGGACTTCATTG	Deletion of *lon* gene (this study)

*References are as in the main text
