# Supplementary material for: Activation of a Cell Surface Signaling Pathway in Pseudomonas aeruginosa Requires ClpP Protease and New Sigma Factor Synthesis
Source: Front Microbiol. 2017 Dec 12;8:2442. doi: 10.3389/fmicb.2017.02442 (PMC5733041; doi:10.3389/fmicb.2017.02442)
Supplement: Supplementary file 2 [file Image_1.pdf]

Supplementary Figures

Activation of a Cell Surface Signaling Pathway in *Pseudomonas aeruginosa* requires ClpP Protease and New Sigma Factor Synthesis

Thomas F. Bishop, Lois W. Martin and Iain L. Lamont*

*** Correspondence:** Corresponding Author: iain.lamont@otago.ac.nz

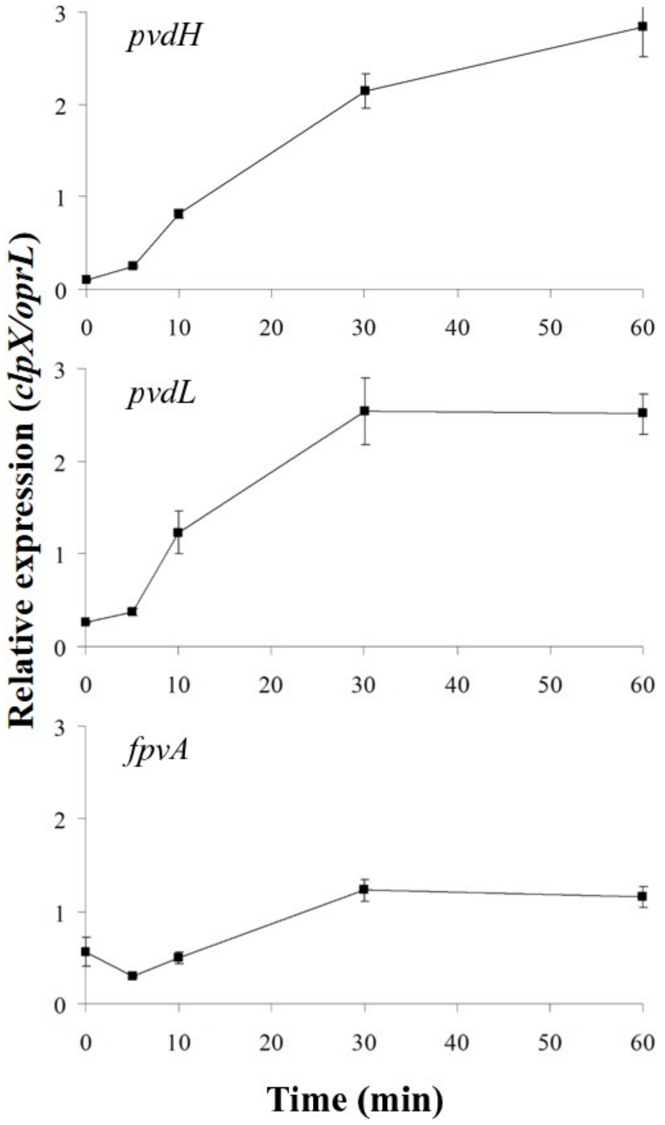

Supplementary Figure S1. Activation of gene expression following addition of pyoverdine. Pyoverdine was added to *P. aeruginosa* PAO1 *pvdF* bacteria (0 minutes) and samples were collected at intervals and analyzed by RT-qPCR. The amounts of *pvdH*, *pvdL* and *fpvA* transcripts are shown relative to the reference genes *clpX* and *oprL*. Data are means of six technical replicates with standard deviation shown. Similar results were obtained in a replicate experiment (Fig. 2 in the main text).

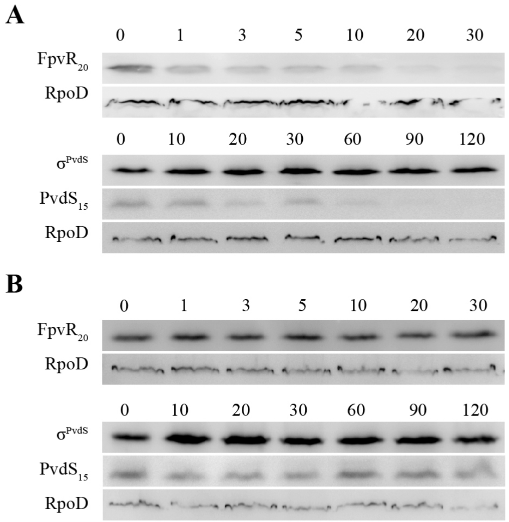

Supplementary Figure S2. Time course of degradation of FpvR and σ^{PvdS} following addition of pyoverdine. Pyoverdine was added to *P. aeruginosa* bacteria (0 minutes). Samples were collected at intervals and analyzed by Western blotting with antibodies against $FpvR_{20}$, σ^{PvdS} or RpoD (loading control). $FpvR_{20}$, PvdS23 (full-size), $PvdS_{15}$ and RpoD are indicated. Times are shown in minutes. A. PAO1 *pvdF* B. PAO1 *pvdF fpvA*. Similar results were obtained in a replicate experiment (Fig. 3 in the main text).

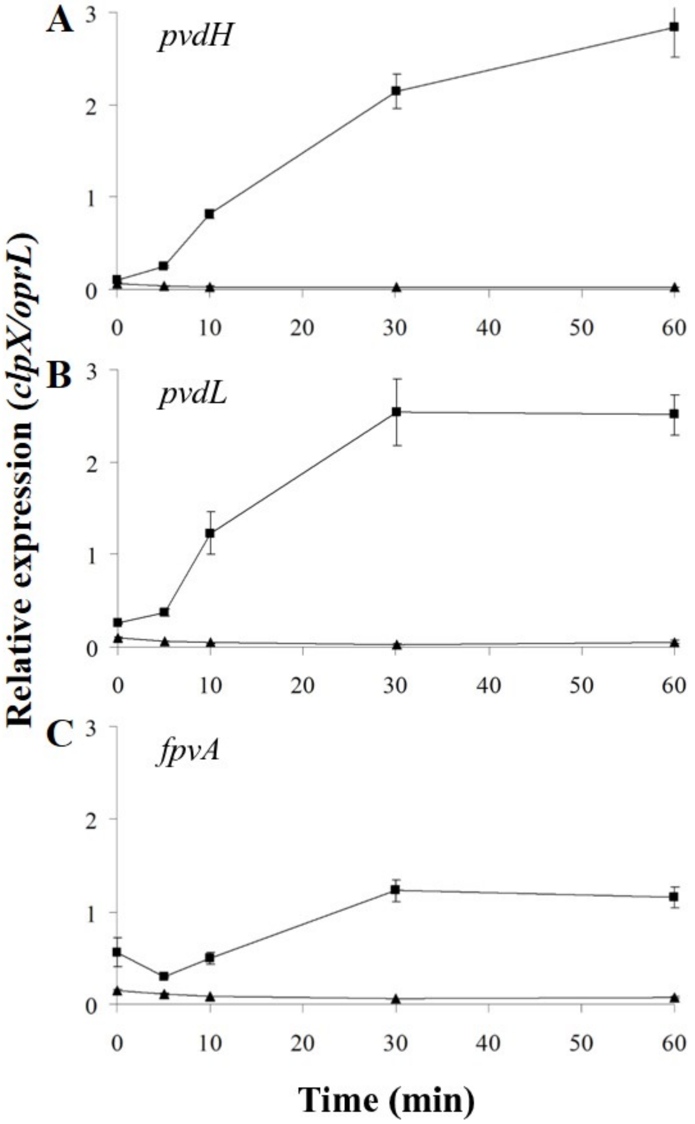

Supplementary Figure S3. Effect of *clpP* mutation on induction of gene expression. Pyoverdine was added (0 minutes) to *P. aeruginosa* PAO1 *pvdF clpP* (black triangles). Samples were collected at intervals and analyzed by RT-qPCR. Data are means of six technical replicates with standard deviation shown. Equivalent data from strain PAO1 *pvdF* bacteria (Fig. S1) (black squares) are included for comparison. A. *pvdH*. B. *pvdL*. C. *fpvA*. Similar results were obtained in a replicate experiment (Fig. 4 in the main text).

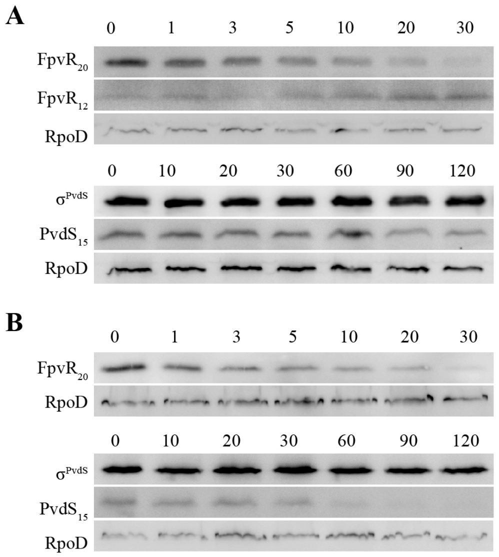

Supplementary Figure S4. Effects of *clpP* mutation on degradation of FpvR$_{20}$ and σ^{PvdS}. Pyoverdine was added (0 minutes) to *P. aeruginosa* PAO1 *pvdF* bacteria containing a mutation in the *clpP* gene. Samples were collected at intervals and analyzed by Western blotting. Times are shown in minutes. Times are shown in minutes. A. *P. aeruginosa* PAO1 *pvdF clpP*. B. *P. aeruginosa* PAO1 *pvdF clpP* (minictx::*tig*-*clpP*). FpvR$_{20}$, FpvR$_{12}$, PvdS23 (full-size), PvdS$_{15}$ and RpoD are indicated. FpvR$_{12}$ was not detected in *P. aeruginosa* PAO1 *pvdF clpP* (minictx::*tig*-*clpP*). Similar results were obtained in a replicate experiment (Fig. 5 in the main text).

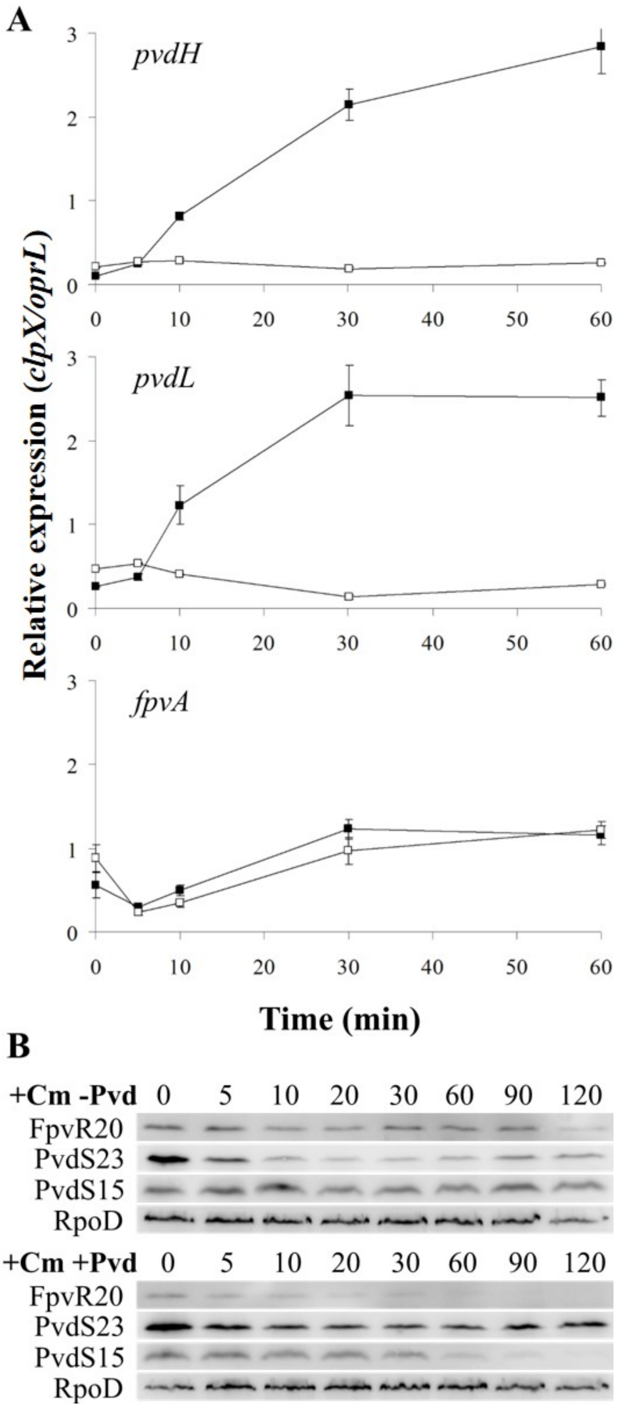

Supplementary Figure S5. Effect of chloramphenicol on induction of gene expression. Chloramphenicol (Cm) and then pyoverdine were added to *P. aeruginosa* PAO1 *pvdF* bacteria (0 minutes) and samples were collected at intervals. A. Samples were analyzed by RT-qPCR for *pvdH*, *pvdL*, and *fpvA*. Open squares, Cm present; Black squares, Cm absent. Data are means of six technical replicates with standard deviation shown. B. Samples were analyzed by Western blotting for σ^{PvdS} or $FpvR_{20}$ in the presence or absence of pyoverdine, as shown. $FpvR_{20}$, σ^{PvdS} (full-size), $PvdS_{15}$ and RpoD are indicated. Times are shown in minutes. Similar results were obtained in a replicate experiment (Fig. 6 in the main text).

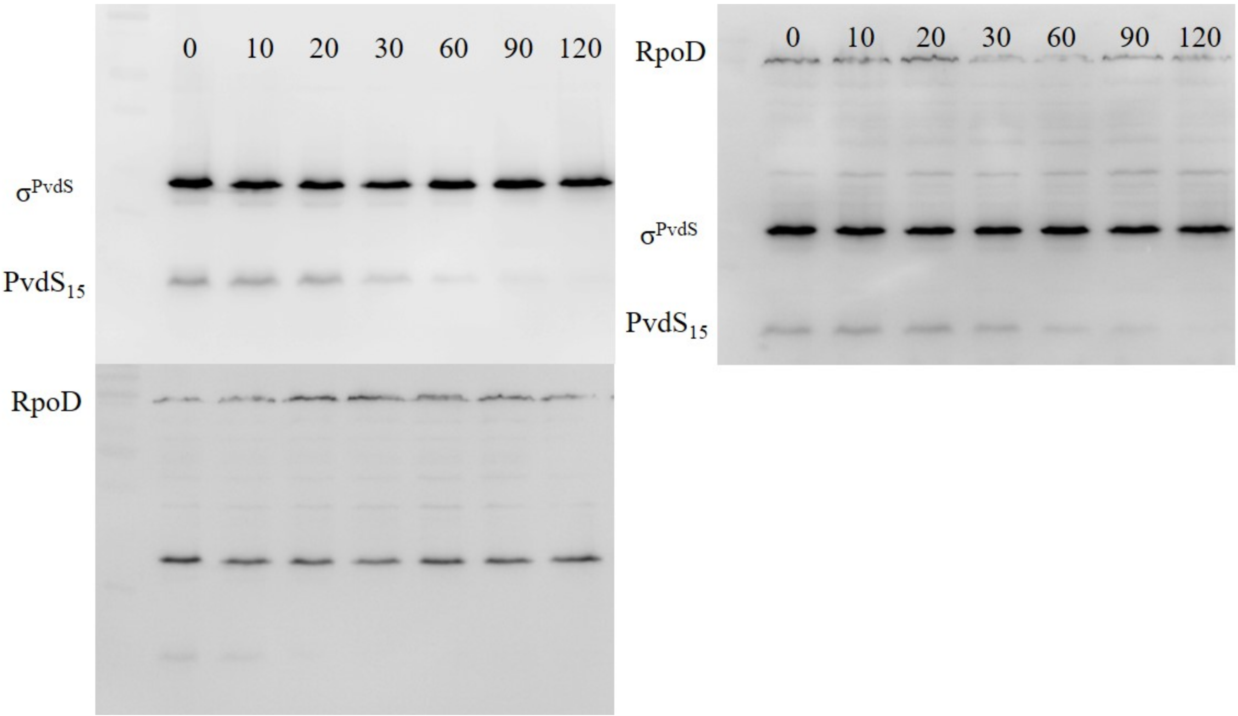

Supplementary Figure S6. A *lon* mutation does not affect degradation of σ^{PvdS}.
Pyoverdine was added (0 minutes) to *P. aeruginosa* PAO1 *pvdF* bacteria containing a mutation in the *lon* gene. Samples were collected at intervals and analyzed by Western blotting. Times are shown in minutes. The positions of σ^{PvdS}, $PvdS_{15}$ and RpoD are indicated. Two replicate experiments are shown, with two exposures shown in the left panel.
