# Supplementary material for: Activation of a Cell Surface Signaling Pathway in Pseudomonas aeruginosa Requires ClpP Protease and New Sigma Factor Synthesis
Source: Front Microbiol. 2017 Dec 12;8:2442. doi: 10.3389/fmicb.2017.02442 (PMC5733041; doi:10.3389/fmicb.2017.02442)
Supplement: Supplementary file 3 [file Image_2.PDF]

Supplementary Data

Activation of a Cell Surface Signaling Pathway in *Pseudomonas aeruginosa* requires ClpP Protease and New Sigma Factor Synthesis

Thomas F. Bishop, Lois W. Martin and Iain L. Lamont*

*** Correspondence:** Corresponding Author: iain.lamont@otago.ac.nz

This file comprises full scans of the entire original Western blots that were used to derive the cropped images included in the manuscript and in the supplementary figures. Figure numbers and detected proteins are indicated.

Fig. 3

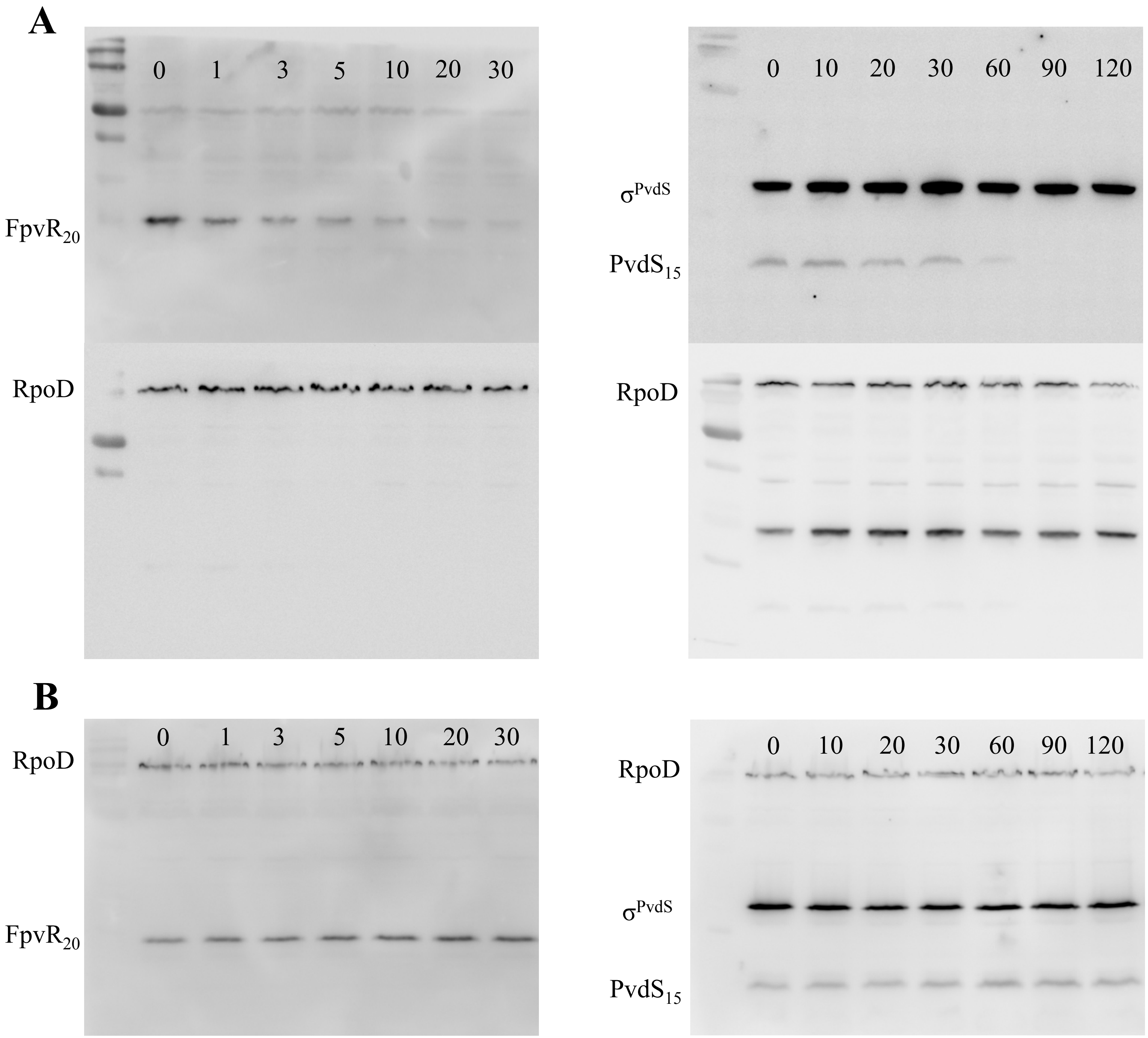
A
0 1 3 5 10 20 30
FpvR20
RpoD
0 10 20 30 60 90 120
σPvdS
PvdS15
RpoD
B
0 1 3 5 10 20 30
RpoD
FpvR20
0 10 20 30 60 90 120
RpoD
σPvdS
PvdS15

Fig. 5

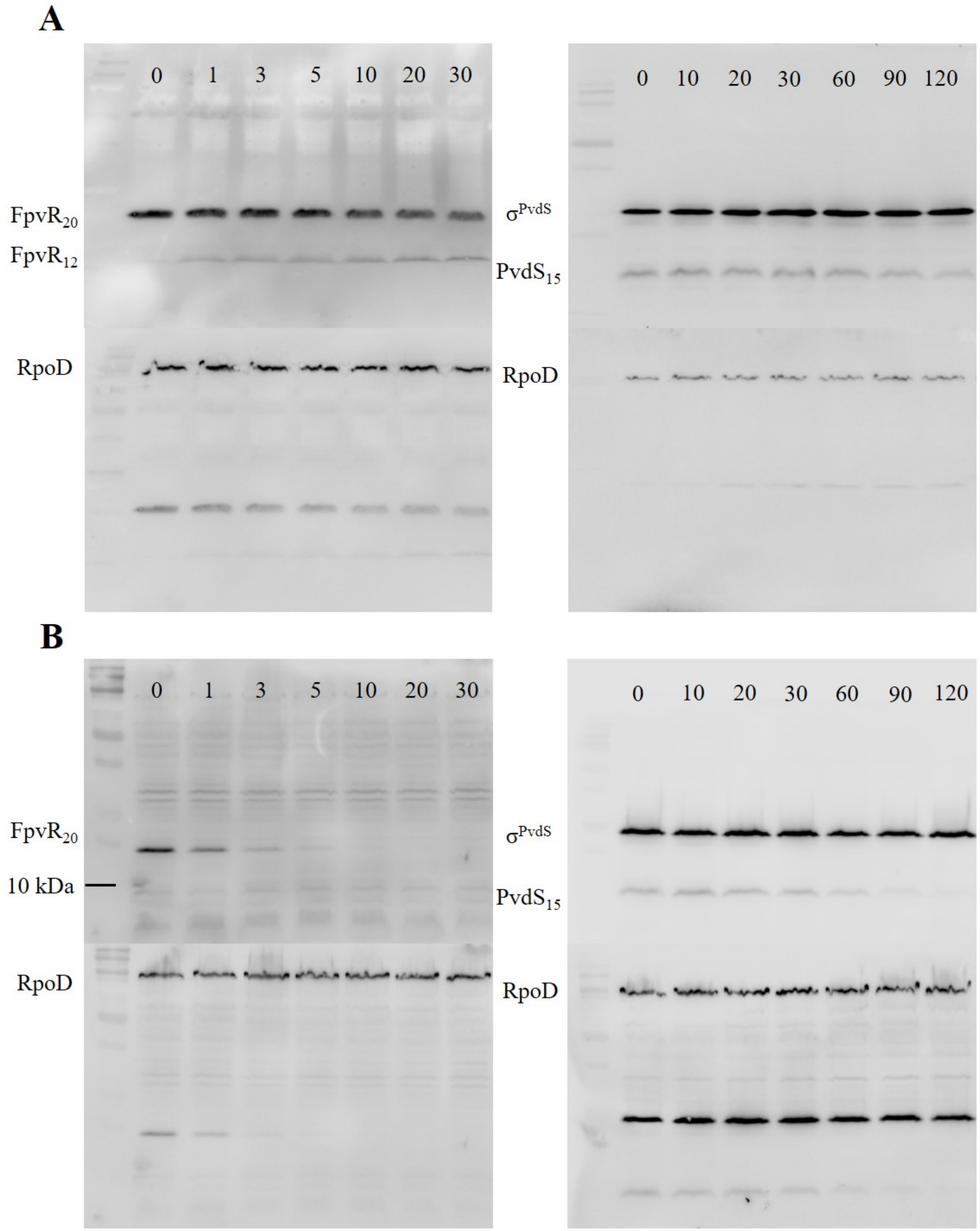
A
0 1 3 5 10 20 30
FpvR20
FpvR12
RpoD
0 10 20 30 60 90 120
σPvdS
PvdS15
RpoD
B
0 1 3 5 10 20 30
FpvR20
10 kDa
RpoD
0 10 20 30 60 90 120
σPvdS
PvdS15
RpoD

Fig. 6B

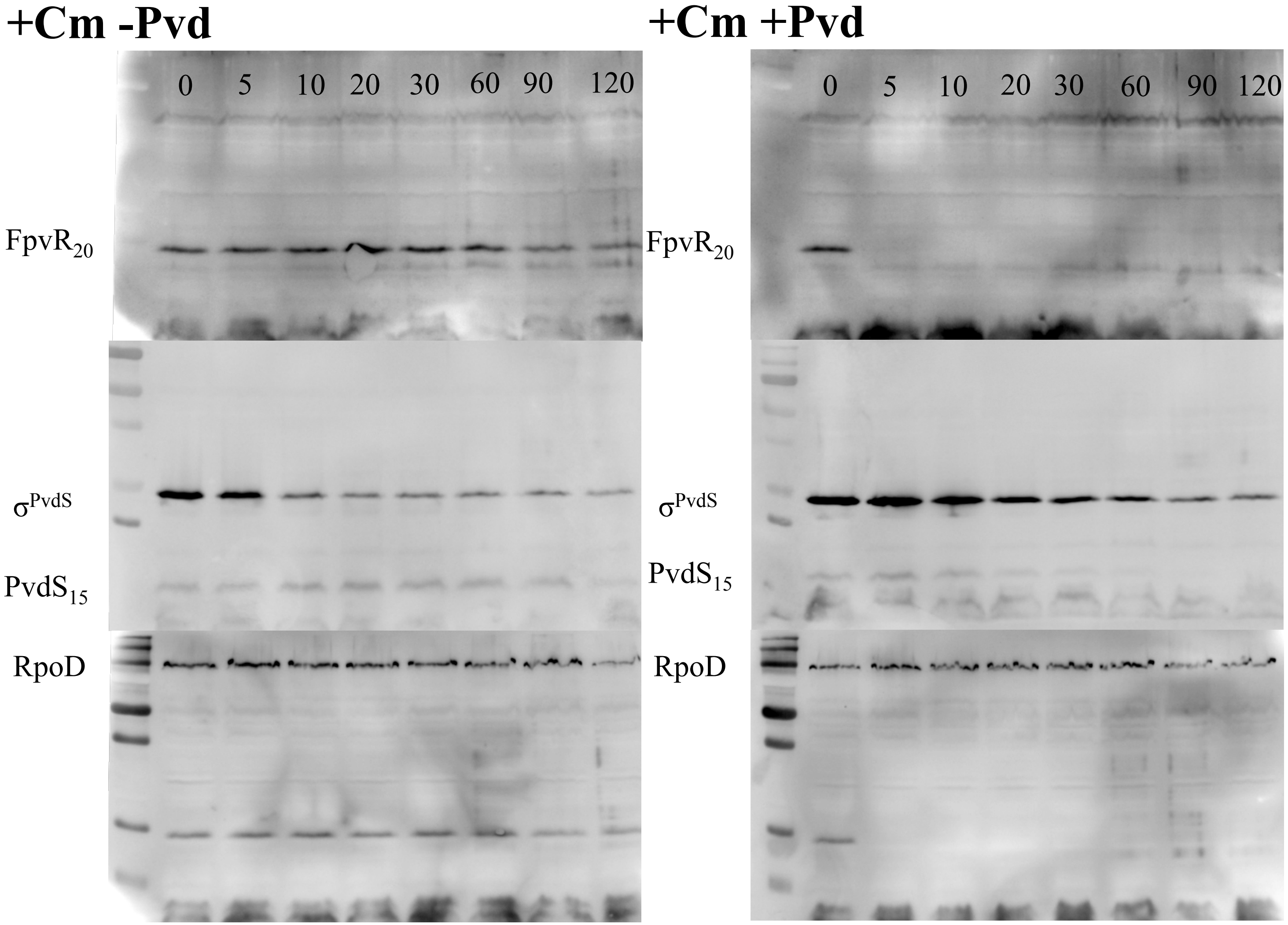
+Cm -Pvd
0
5
10
20
30
60
90
120
FpvR$_{20}$
σ^{PvdS}
PvdS$_{15}$
RpoD
+Cm +Pvd
0
5
10
20
30
60
90
120
FpvR$_{20}$
σ^{PvdS}
PvdS$_{15}$
RpoD

Fig. S2

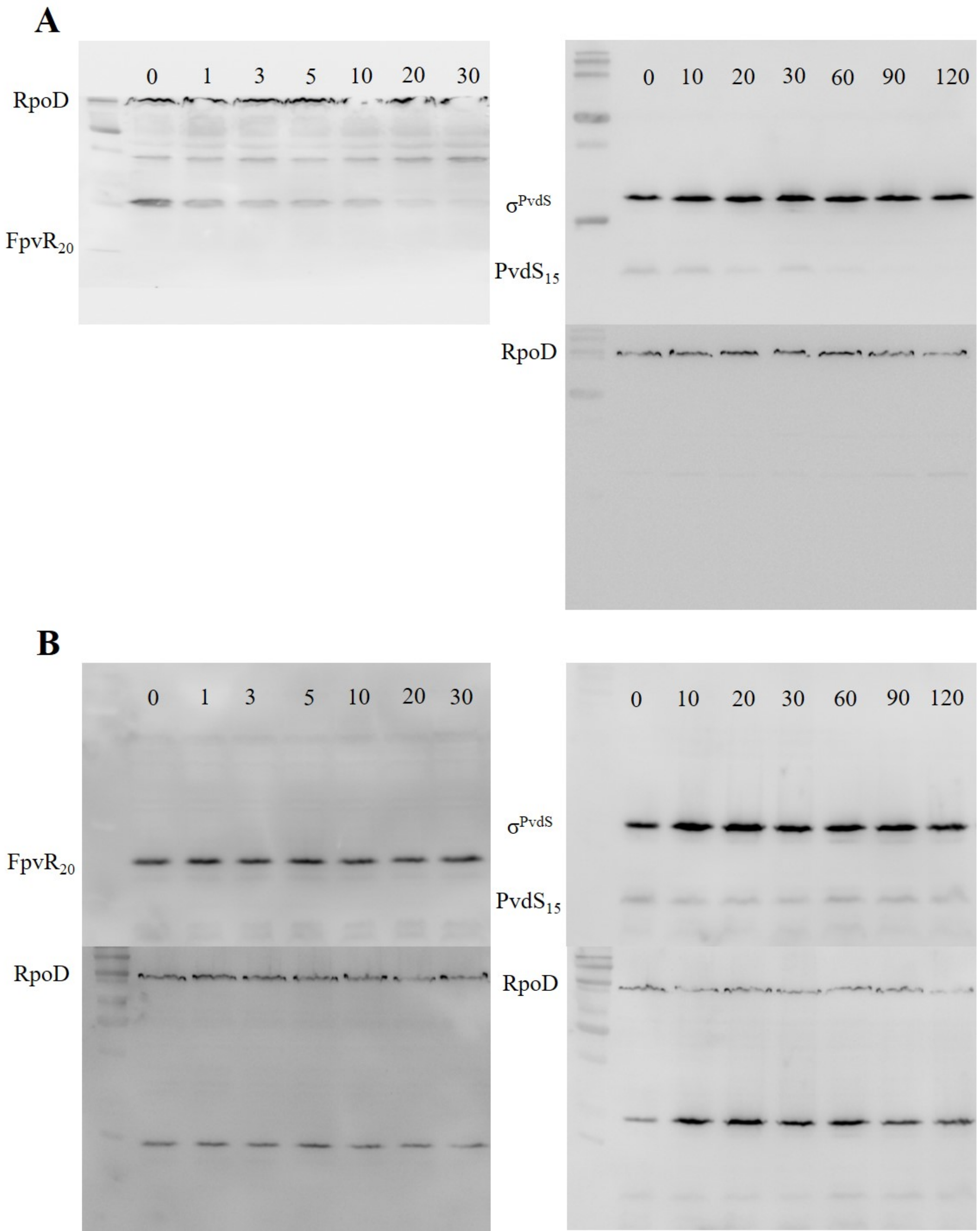
A
0 1 3 5 10 20 30
RpoD
FpvR20
0 10 20 30 60 90 120
σPvdS
PvdS15
RpoD
B
0 1 3 5 10 20 30
FpvR20
RpoD
0 10 20 30 60 90 120
σPvdS
PvdS15
RpoD

Fig. S4

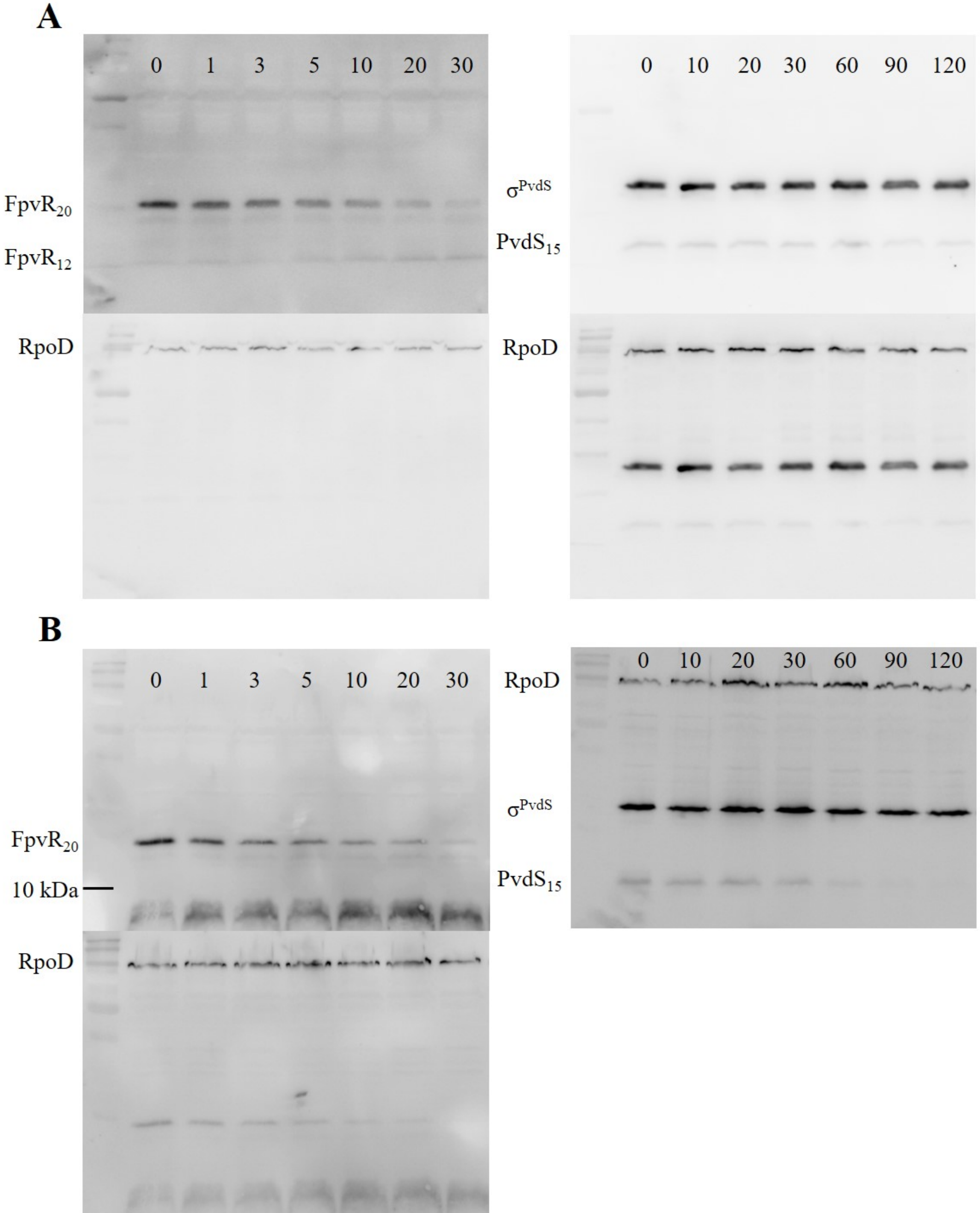

A
0
1
3
5
10
20
30
FpvR20
FpvR12
RpoD
0
10
20
30
60
90
120
σPvdS
PvdS15
RpoD
B
0
1
3
5
10
20
30
FpvR20
10 kDa
RpoD
0
10
20
30
60
90
120
RpoD
σPvdS
PvdS15

Fig. S5B

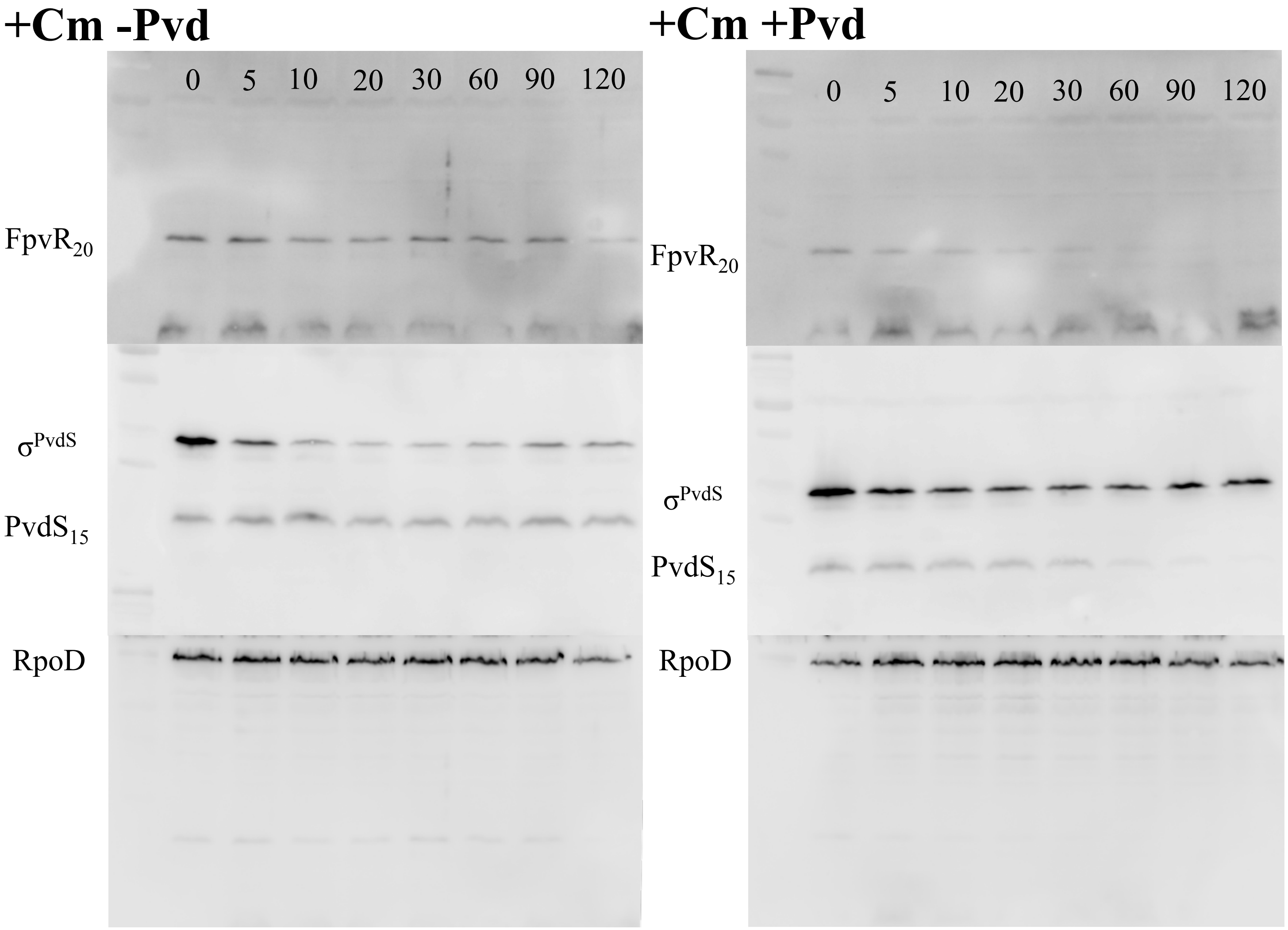
+Cm -Pvd
+Cm +Pvd
0 5 10 20 30 60 90 120
0 5 10 20 30 60 90 120
FpvR20
FpvR20
σPvdS
σPvdS
PvdS15
PvdS15
RpoD
RpoD
